# Un cas de COVID-19 compliqué d´embolie avec deux tests PCR initialement négatifs malgré des signes scanographiques

**DOI:** 10.11604/pamj.supp.2020.35.2.24590

**Published:** 2020-07-01

**Authors:** Ibrahima Niang, Daouda Thioub, Ibrahima Diallo, Joseph Coumba Ndoffene Diouf, Khadija Ndiaye Diouf, Sokhna Ba

**Affiliations:** 1 Service d´imagerie médicale Chnu (Centre Hospitalier National Universitaire) de Fann, Dakar Senegal; 2 Service des maladies infectieuses et tropicales Chnu de Fann, Dakar Senegal

**Keywords:** COVID-19, PCR, embolie, verre dépoli, tomodensitométrie

## Abstract

La maladie à coronavirus 2019 (COVID-19) déclarée en Chine en fin 2019 s´est rapidement généralisée aux autres continents. Son diagnostic se fait par test PCR (Polymerase Chain Reaction) sur des prélèvements naso-pharyngés. Ce test bien que spécifique est d´une sensibilité moindre comparé à la TDM thoracique. Nous rapportons le cas d´un patient testé négatif à deux reprises et chez qui la TDM retrouvait des signes typiques de COVID-19 et une embolie pulmonaire. Et ce n´est qu´après un troisième test PCR qu´il a été positif. Ce qui montre l´intérêt de répéter plusieurs fois les tests PCR mais également de considérer les signes scanographiques comme argument diagnostic devant induire une prise en charge adéquate.

## Introduction

La COVID-19 déclarée à Wuhan en chine depuis la fin du mois de décembre 2019 s´est rapidement propagée sur le reste du monde et au Sénégal le premier cas a été rapporté le 02/03/ 2020 [1]. La méthode diagnostique de référence est la recherche en laboratoire d’ARN viral par PCR à partir d’écouvillonnages nasopharynge’s. Cependant, l’obtention des résultats demande plusieurs heures, et seuls certains laboratoires disposent de ce test. Par ailleurs, si la spécificité du test viral est excellente, sa sensibilité est imparfaite (60 à 70 %) car dépend de la qualité du prélèvement et du taux de réplication virale au sein des voies aériennes respiratoires supérieures [2,3] . De ce fait la TDM est indiquée comme moyen diagnostic en présence de symptomatologie avec test PCR négatif, de même comme moyen de suivi des patients positifs avec décompensation respiratoire à la recherche de complications [4,5]. La pathologie thromboembolique étant parmi les complications les plus fréquentes et les plus péjoratives de cette infection [6].

## Patient et observation

**Figure 1:**
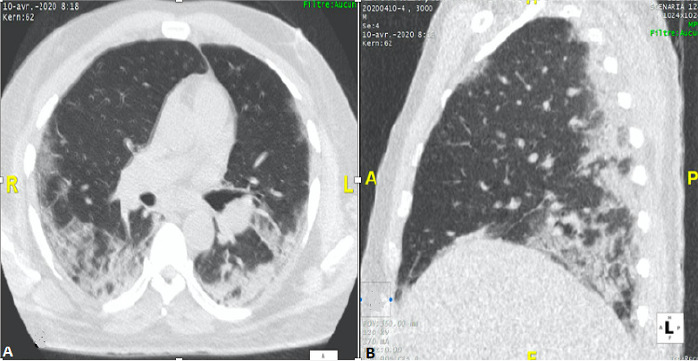
TDM thoracique en fenêtre pulmonaire. (A) coupe axiale, montrant des lésions sous pleurales faites d´opacités en verre dépoli et de condensation; (B) reconstruction sagittale, démontre la prédominance postéro-basale

**Figure 2:**
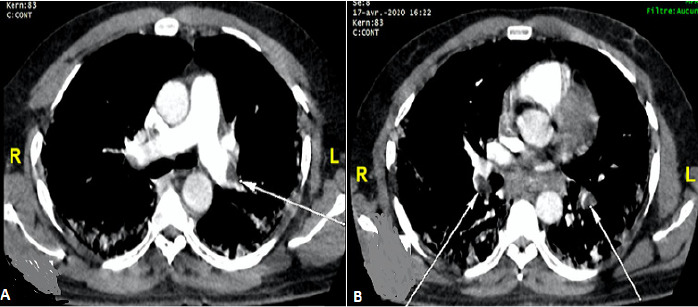
TDM thoracique avec injection de produit de contraste en coupe axiale fenêtre médiastinale. (A) défect endoluminal marginal de l´artère pulmonaire lobaire inferieur gauche (flèche blanche); (B) défect endoluminal bilatéral des artères segmentaires postérieurs (flèches blanches)

**Figure 3:**
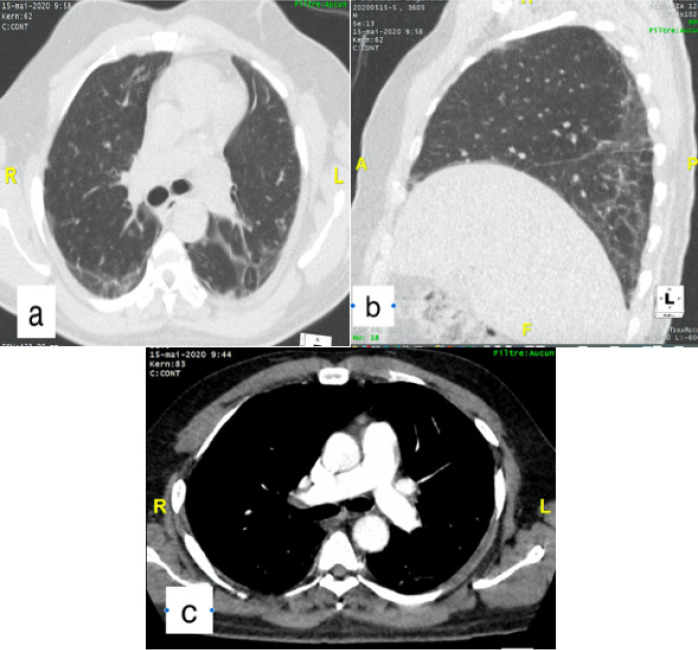
TDM thoracique avec injection de produit de contraste. (A) coupe axiale en fenêtre parenchymateuse, montrant la régression de la condensation laissant place à de discrètes opacités sous pleural en verre dépoli; (B) fenêtre pulmonaire reconstruction sagittale, démontre aussi la régression des lesions; (C) fenêtre médiastinale perméabilisation des artères pulmonaires

Nous rapportons le cas d´un patient de 58 ans sans comorbidité connu qui a consulté aux urgences pour une symptomatologie faite de dyspnée, myalgie et sensation de fatigue évoluant depuis une semaine. Dans ce contexte épidémique, un premier test PCR sur écouvillonnage naso-pharyngé a été réalisé avec un résultat négatif. Une TDM thoracique sans injection de produit de contraste réalisée le lendemain a permis de mettre en évidence des plages de verre dépoli associées à des foyers de condensation bilatérales, exclusivement sous pleurales et prédominant au niveau postéro-basal (Figure 1). Le patient est resté par la suite confiné chez lui après un deuxième test PCR encore négatif. Une semaine plus tard devant l´aggravation de la dyspnée, une seconde TDM thoracique réalisée cette fois avec injection de produit de contraste a permis de mettre en évidence des lésions parenchymateuses superposables à celles déjà décrites associées à une embolie pulmonaire bilatérale lobaire à gauche et segmentaire à droite (Figure 2). Devant cette embolie pulmonaire il a été pris en charge au service de cardiologie et un troisième test PCR réalisé est revenu positif. Ainsi il a été admis au CTE (centre de traitement épidémiologique) de l´hôpital de FANN où il a reçu un traitement anticoagulant à dose curative et le protocole de traitement COVID-19 composé d´hydroxychloroquine (200mg 1cpx3/jr pendant 10 jr) et d´azythromycine (500mg 1cp/jr pendant 03 jr). Son état clinique s´est amélioré sous ce traitement et au bout de 12 jours il est sorti d´hôpital après deux tests PCR successifs négatifs. Une tomodensitométrie de contrôle avec injection de produit de contraste réalisée après sa sortie d´hôpital a mis en évidence une régression des lésions pulmonaires et une perméabilisation des artères pulmonaires (Figure 3).

## Discussion

Du fait de son niveau de transmissibilité, il est crucial de diagnostiquer, d´isoler et de traiter les patients infectés à la COVID-19 pour éviter à la fois la diffusion du virus et la survenue de complications [7]. Le test PCR sur prélèvement naso-pharyngé est à ce jour l´examen de référence pour le diagnostic du COVID-19 avec une bonne spécificité mais une sensibilité moindre [8]. Cette faible sensibilité est à l´origine de faux négatifs pouvant être dû au kit de test, à la technique de prélèvement, à la cinétique non linéaire du virus entre autres [3]. La faible sensibilité du test PCR devrait être compensée par la répétition des tests et l´utilisation de la TDM thoracique qui présente une meilleure sensibilité [2,9]. La TDM présente également l´intérêt d´être disponible, de réalisation rapide et non invasive. Par ailleurs en plus des signes en faveur de COVID-19 essentiellement représenté par les opacités en verre dépoli souvent bilatéral de localisation sous pleurale, elle permet aussi de rechercher des complications intercurrentes à type d´embolie pulmonaire ou autres [10]. Quant à la distinction au scanner avec d´autres pneumopathies notamment virales, il est établi que la localisation sous pleurale du verre dépoli est un bon signe discriminant [11]. Ainsi un patient symptomatique avec des signes TDM de Covid-19 devrait être pris en charge comme un patient Covid, ce qui n´est pas encore le cas dans la stratégie nationale de riposte sénégalaise. Le risque de diffusion du virus étant ainsi majoré de même que la survenue d´éventuelles complications La fréquence des complications thromboemboliques a été rapportée par des auteurs et serait dû à une réaction inflammatoire excessive en réponse à l´infection virale [12]. Ce qui justifie un traitement anticoagulant préventif d´autant plus s´il existe d´autres facteurs de risque thromboembolique.

## Conclusion

Cette observation démontre que la faible sensibilité du test PCR devrait amener à répéter les tests négatifs chez les patients symptomatiques avec test PCR initialement négatif et images scanographiques évocatrices; et aussi utiliser la TDM thoracique comme argument diagnostic dans ces cas. Cette TDM devrait être également à la recherche d´embolie pulmonaire d´autant plus que les données de la littérature suggèrent un haut potentiel thrombogène de cette infection.

## Conflits d’intérêts

Les auteurs ne déclarent aucun conflit d´intérêts.
